# Host immune status-specific production of gliotoxin and bis-methyl-gliotoxin during invasive aspergillosis in mice

**DOI:** 10.1038/s41598-017-10888-9

**Published:** 2017-09-08

**Authors:** Janyce A. Sugui, Stacey R. Rose, Glenn Nardone, Muthulekha Swamydas, Chyi-Chia R. Lee, Kyung J. Kwon-Chung, Michail S. Lionakis

**Affiliations:** 1 Molecular Microbiology Section, Laboratory of Clinical Immunology & Microbiology (LCIM), National Institute of Allergy & Infectious Diseases (NIAID), National Institutes of Health (NIH), Bethesda, MD USA; 2 Fungal Pathogenesis Unit, LCIM, NIAID, NIH, Bethesda, MD USA; 3 Research Technology Branch, NIH, Rockville, MD USA; 4 Laboratory of Pathology, Center for Cancer Research, National Cancer Institute (NCI), NIH, Bethesda, MD USA; 5 Present Address: Division of Infectious Diseases, Baylor College of Medicine, Houston, Texas USA

## Abstract

Delayed diagnosis in invasive aspergillosis (IA) contributes to its high mortality. Gliotoxin (GT) and bis-methyl-gliotoxin (bmGT) are secondary metabolites produced by *Aspergillus* during invasive, hyphal growth and may prove diagnostically useful. Because IA pathophysiology and GT’s role in virulence vary depending on the underlying host immune status, we hypothesized that GT and bmGT production *in vivo* may differ in three mouse models of IA that mimic human disease. We defined temporal kinetics of GT and bmGT in serum, bronchoalveolar lavage fluid (BALF) and lungs of *A. fumigatus*-infected chronic granulomatous disease (CGD), hydrocortisone-treated, and neutropenic mice. We harvested lungs for assessment of fungal burden, histology and GT/bmGT biosynthetic genes’ mRNA induction. GT levels were higher in neutropenic versus CGD or steroid-treated lungs. bmGT was persistently detected only in CGD lungs. GT, but not bmGT, was detected in 71% of sera and 50% of BALF of neutropenic mice; neither was detected in serum/BALF of CGD or steroid-treated mice. Enrichment of GT in *Aspergillus*-infected neutropenic lung correlated with fungal burden and hyphal length but not induction of GT biosynthetic genes. In summary, GT is detectable in mouse lungs, serum and BALF during neutropenic IA, suggesting that GT may be useful to diagnose IA in neutropenic patients.

## Introduction

Invasive aspergillosis (IA) continues to cause substantial mortality among immunocompromised hosts, despite advances in antifungal therapy^1, 2^. Suboptimal diagnosis contributes to the high mortality of IA^3^. Over the last decade, the diagnosis of IA has evolved from reliance on culture and/or histopathology to the incorporation of biomarkers into the diagnostic algorithms in hopes of an early, accurate and non-invasive means of identifying infection. For instance, detection of galactomannan (GM) or beta-D-glucan (BDG), both fungal cell-wall components, are now considered to be part of the mycological criteria for defining “probable” IA per the European Organization for Research and Treatment of Cancer/Invasive Fungal Infections Cooperative Group and the NIAID Mycoses Study Group (EORTC/MSG) Consensus Group^4^. However, the clinical utility of GM testing has been hindered by false-positive results, such as with administration of certain antibiotics, and false-negatives in patients already receiving mold-active antifungal prophylaxis or therapy^5, 6^. The usefulness of BDG testing in clinical practice has similarly been hindered by suboptimal specificity^7, 8^. Thus far, the clinical utility of molecular diagnostics, such as *Aspergillus* polymerase chain reaction (PCR), has been limited by the lack of assay standardization as well as challenges of interpreting results in the setting of airway fungal colonization or laboratory contamination^3, 4^. Other non-culture based diagnostic tools such as lateral flow devices and detection of volatile organic compounds appear promising, but have yet to be validated in the context of widespread clinical practice^9, 10^. Thus, improving the diagnosis of IA via the implementation of new and reliable biomarkers remains an important area of investigation.

GT is a fungal secondary metabolite which was originally isolated from *Trichoderma* species and is now widely used in the agricultural industry to protect crops by controlling pathogenic fungal infections^11^. GT is also produced by *Aspergillus* species, particularly *Aspergillus fumigatus*, which remains the most common cause of IA among immunocompromised hosts^2^. Several characteristics make GT an appealing biomarker for the diagnosis of IA: 1) GT is produced by several pathogenic species of *Aspergillus* ^12^ but not by other common causes of invasive fungal infections such as *Candida* species^13^; 2) GT is produced only by the hyphal forms of *Aspergillus* which are found in tissue during invasive disease, but not by the conidial developmental program of the fungus, which is present in the airways during colonization^14^; and 3) GT has been identified in mouse lung and serum following experimental infection, and, retrospectively, in serum samples from humans with suspected IA^15^.

GT is also of clinical interest because of its biological significance as a fungal virulence factor, via a variety of potential immunosuppressive mechanisms^16, 17^. Notably, the significance of GT as a virulence factor appears to vary depending on the immune status of the host; for instance, *Aspergillus* strains rendered unable to produce GT via deletion of the *gliP* gene demonstrate attenuated virulence in non-neutropenic mice, while their virulence in neutropenic mice is maintained^18, 19^. Thus, host immunity is a relevant consideration in the study of GT as a potential diagnostic biomarker for IA.

The biological activity of GT is thought to be at least partly due to its disulfide bond, which may interfere with neutrophil NADPH oxidase^20^. Methylation of the disulfide bond results in the formation of a related fungal metabolite, bmGT, which has been shown to downregulate GT synthesis* in vitro* ^21^. Recently, bmGT has been detected in serum and bronchoalveolar lavage fluid (BALF) from some humans with suspected IA^22, 23^. Thus, bmGT, in addition to GT, may harbor potential as a clinically useful diagnostic biomarker for IA.

However, clinical investigations to date regarding the diagnostic utility of GT and bmGT have relied primarily on retrospective associations in small numbers of human samples; there remains a paucity of data regarding the temporal and spatial dynamics of GT and bmGT production by *Aspergillus* in the setting of acute infection. Specifically, prior studies lack information regarding the timing and location of GT and bmGT production following the development of IA and whether these biomarkers may be differentially expressed depending on the underlying host immune status.

Therefore, in this study, we sought to detect and analyze the kinetics of GT and bmGT in the lungs, BALF and serum of three mouse models of IA that mimic human risk factors for aspergillosis: chronic granulomatous disease (CGD), steroid-induced immunosuppression and neutropenia. Understanding the spatiotemporal dynamics of GT and bmGT in different tissues of various hosts is an important step towards determining the potential utility of such metabolites for clinical diagnosis.

## Results

### CGD, corticosteroid treatment and neutropenia result in differential lung histopathology of *Aspergillus fumigatus*-infected mice

**Figure 1:**
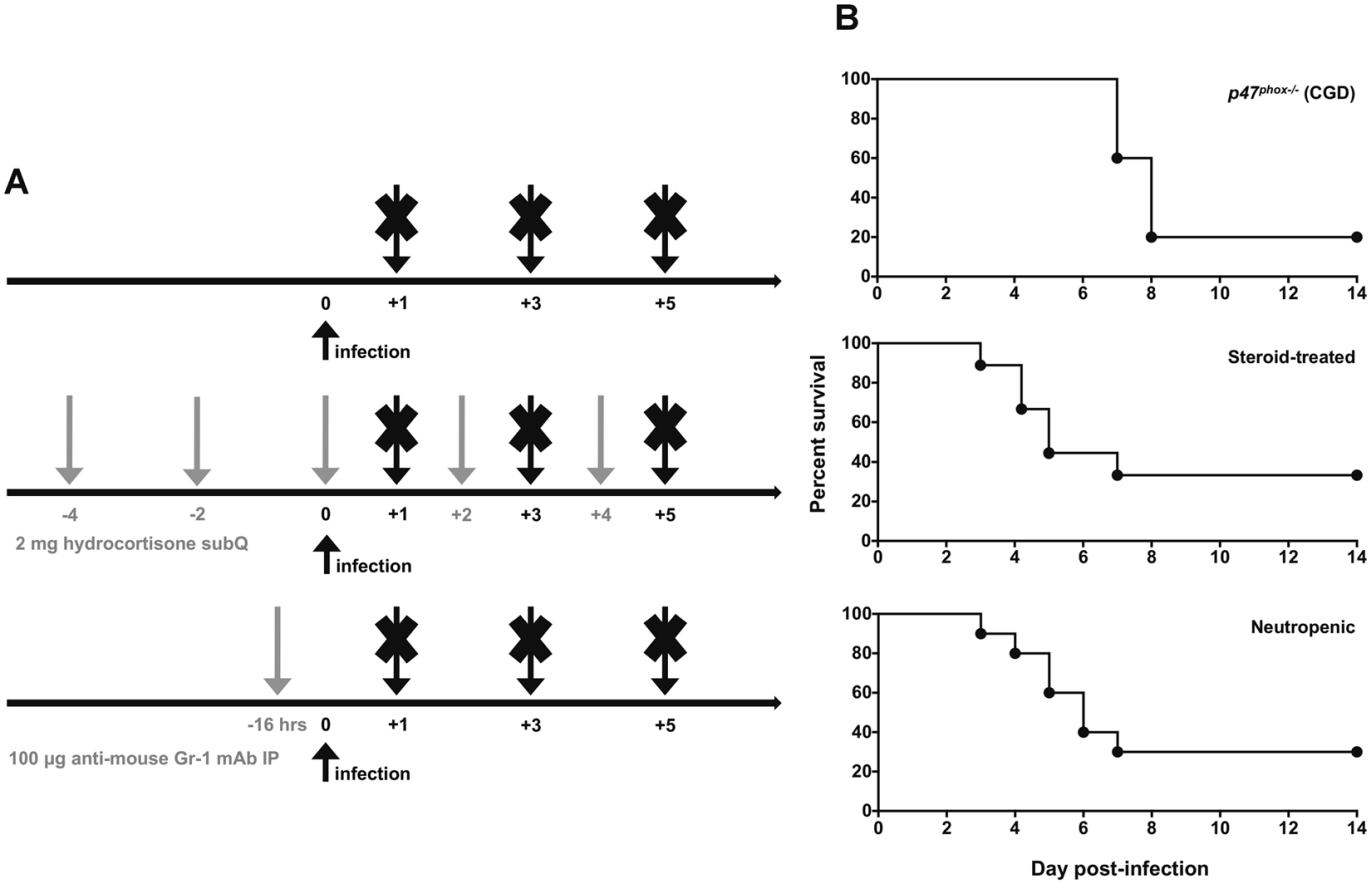
Mouse models of IA. (**A**) Schematic representation of the three mouse models of IA that were used in the present study: *p47* ^*phox−/−*^ CGD mice (upper panel), hydrocortisone-treated C57BL/6 mice (middle panel), and neutropenic C57BL/6 mice (lower panel). Grey arrows indicate the times of administration of hydrocortisone or anti-mouse Gr-1 monoclonal antibody, and black arrows at day 0 indicate the time of infection with *A. fumigatus* conidia, and black arrows at days 1, 3 and 5 indicate the time of euthanization to harvest lungs, serum and BALF for assessment of GT and bmGT levels, and/or histological analyses, and/or transcriptional analyses and/or tissue fungal burden. SubQ, subcutaneous; IP, intraperitoneal. (**B**) Representative survival curves of *p47* ^*phox−/−*^ CGD mice (upper panel), hydrocortisone-treated mice (middle panel), and neutropenic mice (lower panel) infected with *A. fumigatus* conidia.

In order to determine the impact of the different host risk factors on the temporal dynamics of GT and bmGT production during IA, we infected *p47* ^*ph*^ *°* ^*x−/−*^ CGD mice, hydrocortisone-treated C57BL/6 wild-type (WT) mice, and neutropenic WT mice with *Aspergillus fumigatus* conidia via the intrapharyngeal route, as previously described (Fig. 1A)^24^. We used *A. fumigatus* inocula that resulted in approximately an LD_80_ by day 7–10 post-infection in all three models of IA. To achieve similar mortality rates, the steroid-treated and neutropenic mice required a  similar infecting inoculum of ~6 × 10^6^ conidia, whereas CGD mice required a significantly lower infecting inoculum (~3.5 × 10^4^ conidia) (Fig. 1B).

**Figure 2:**
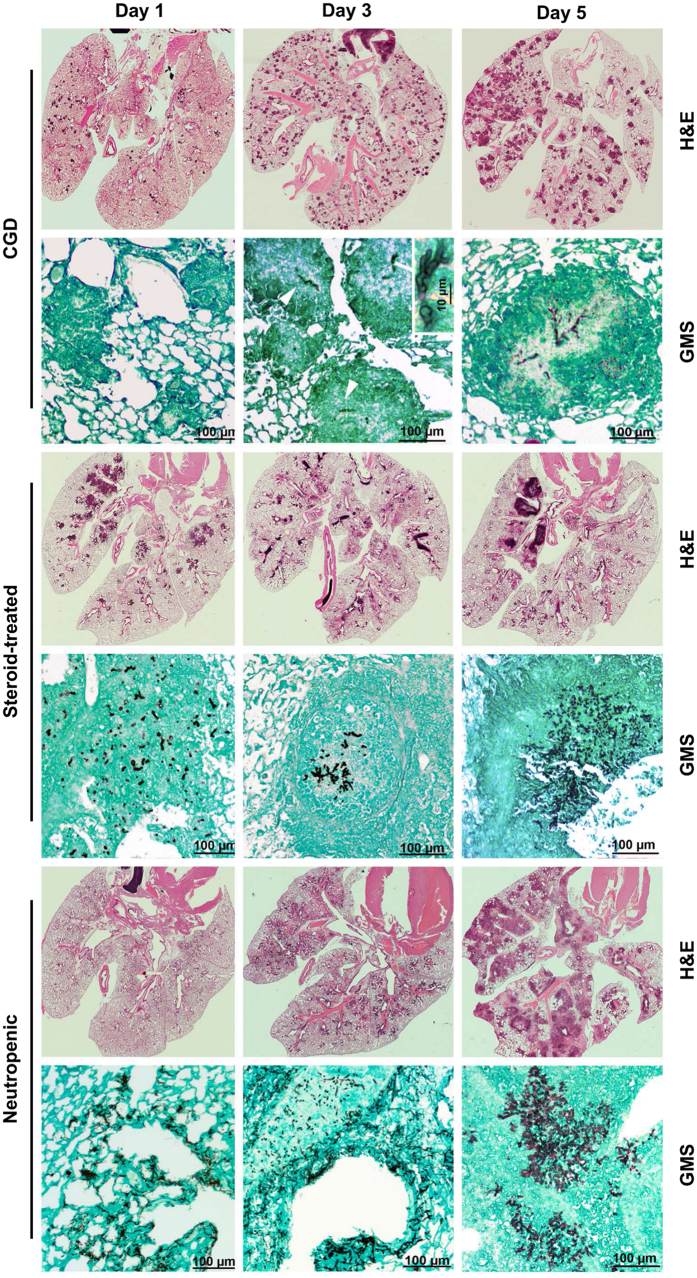
Histological features of IA vary  depending on the underlying host risk factor. Shown are representative H&E and GMS stains from *A. fumigatus*-infected lungs in CGD mice, steroid-treated mice and neutropenic mice at days 1, 3 and 5 post-infection. Magnification, 20x and 200x. Two independent experiments yielded similar results with a total of 4–6 mice per time-point.

Previous studies have demonstrated differential pathogenesis of IA in neutropenic and steroid-treated rabbits. Specifically, abundance of hyphal parenchymal and vascular invasion, coagulative necrosis, and lack of inflammatory infiltrate characterized lesions in neutropenic rabbit lungs, whereas *Aspergillus* lesions in steroid-treated rabbit lungs consisted of phagocyte infiltrates, inflammatory necrosis, and scant hyphal invasion of parenchyma and vasculature^25^. Similar host-specific histopathological features have been observed in *Aspergillus*-infected human lungs^26–28^. Therefore, to systematically delineate the host-specific histopathological features of IA in the three infection models of mice, we euthanized CGD, steroid-treated, and neutropenic animals on days 1, 3, and 5 post-infection and performed histological analyses using hematoxylin and eosin (H&E) and Gomori methenamine silver (GMS) stains (Fig. 2).

**Figure 3:**
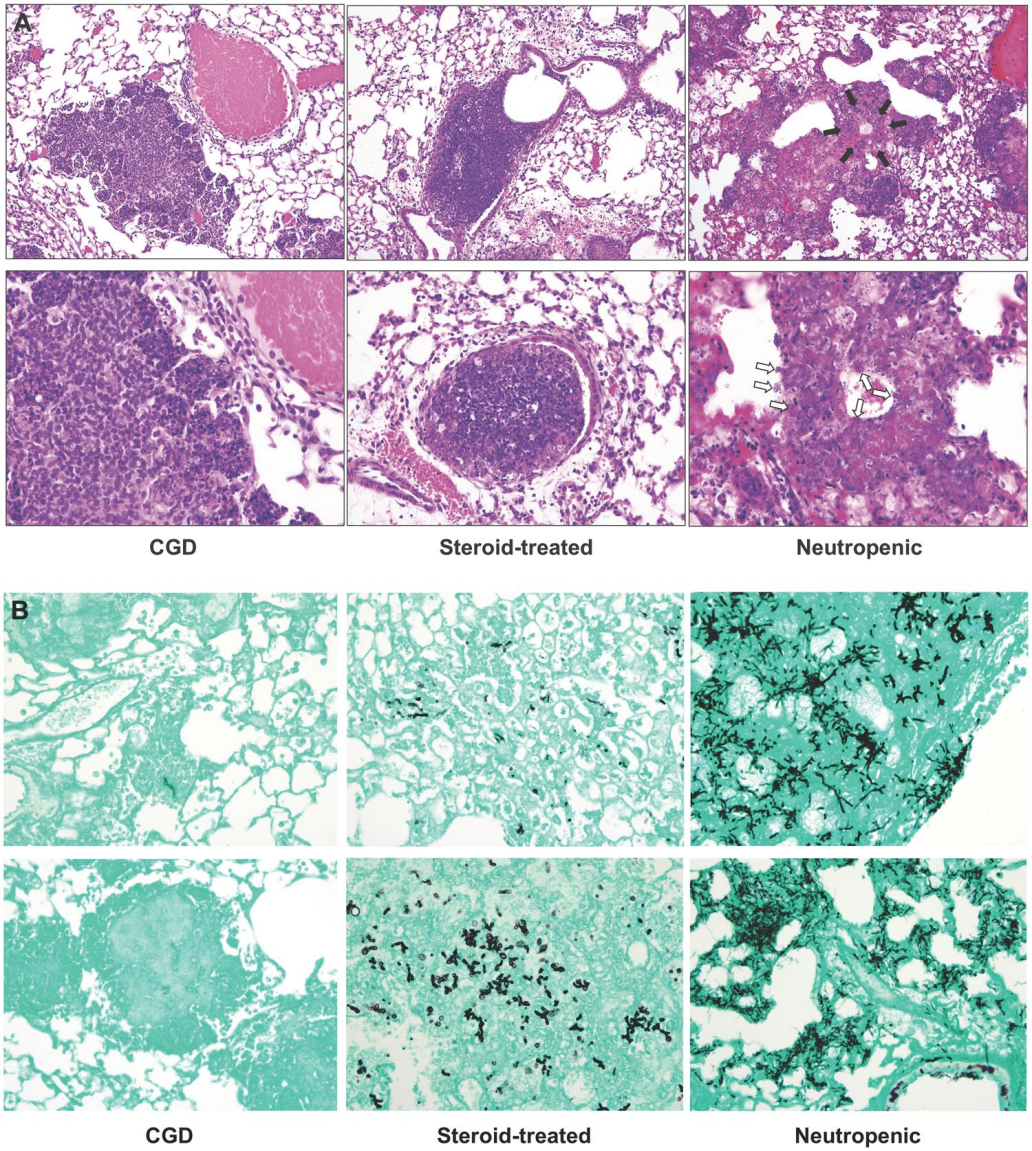
Neutropenic IA results in extensive vascular and peribronchial invasion by *A. fumigatus* hyphae. (**A**) Extensive angioinvasion by *A. fumigatus* hyphae is observed in neutropenic mice but not in CGD or steroid-treated mice. The black arrows indicate the blood vessel at low magnification that was infiltrated by *A. fumigatus* hyphae and the white arrows indicate the *A. fumigatus* hyphae that infiltrate the vessel wall in neutropenic mice. CGD mouse lung images show a non-necrotizing granuloma in the lung parenchyma, with an unaffected adjacent blood vessel. Steroid-treated mouse lung images show phagocytic infiltration with intact adjacent blood vessel (left lower corner of lower panel). 200x (upper panels), 400x (lower panel in steroid-treated mice), 600x (lower panels for CGD and neutropenic mice). Two independent experiments were performed with similar results with a total of 4–6 mice per time-point. (**B**) Peribronchial localization of *A. fumigatus* hyphae is observed in neutropenic mice but not in CGD or steroid-treated mice. Magnification, 400x. Two independent experiments produced similar results with a total of 4–6 mice per time-point.

IA in CGD mice resulted in progressive development of non-necrotizing granulomatous lesions throughout the lung parenchyma, within which *A. fumigatus* hyphae were contained. No angioinvasion or peribronchial invasion by *A. fumigatus* was observed (Figs 2 and 3). Steroid-treated WT mice developed parenchymal lesions consisting of neutrophils and mononuclear cells, within which *A. fumigatus* hyphae were contained; karyorrhexis and inflammatory necrosis were evident. No *Aspergillus* angioinvasion or peribronchial invasion was observed (Figs 2 and 3). Neutropenic mice developed extensive parenchymal lesions characterized by paucity of inflammatory infiltrates until neutrophil recovery occurred by day 5 post-infection. Coagulative tissue necrosis, extensive hyphal vascular invasion and involvement of peribronchial tissues were observed (Figs 2 and 3). Collectively, these findings indicate that, similar to humans and rabbits, the pathogenesis of IA varies greatly in mice depending on the underlying host immune status.

### Accumulation of GT and bmGT in the lungs during IA varies depending on the underlying host risk factor

**Figure 4:**
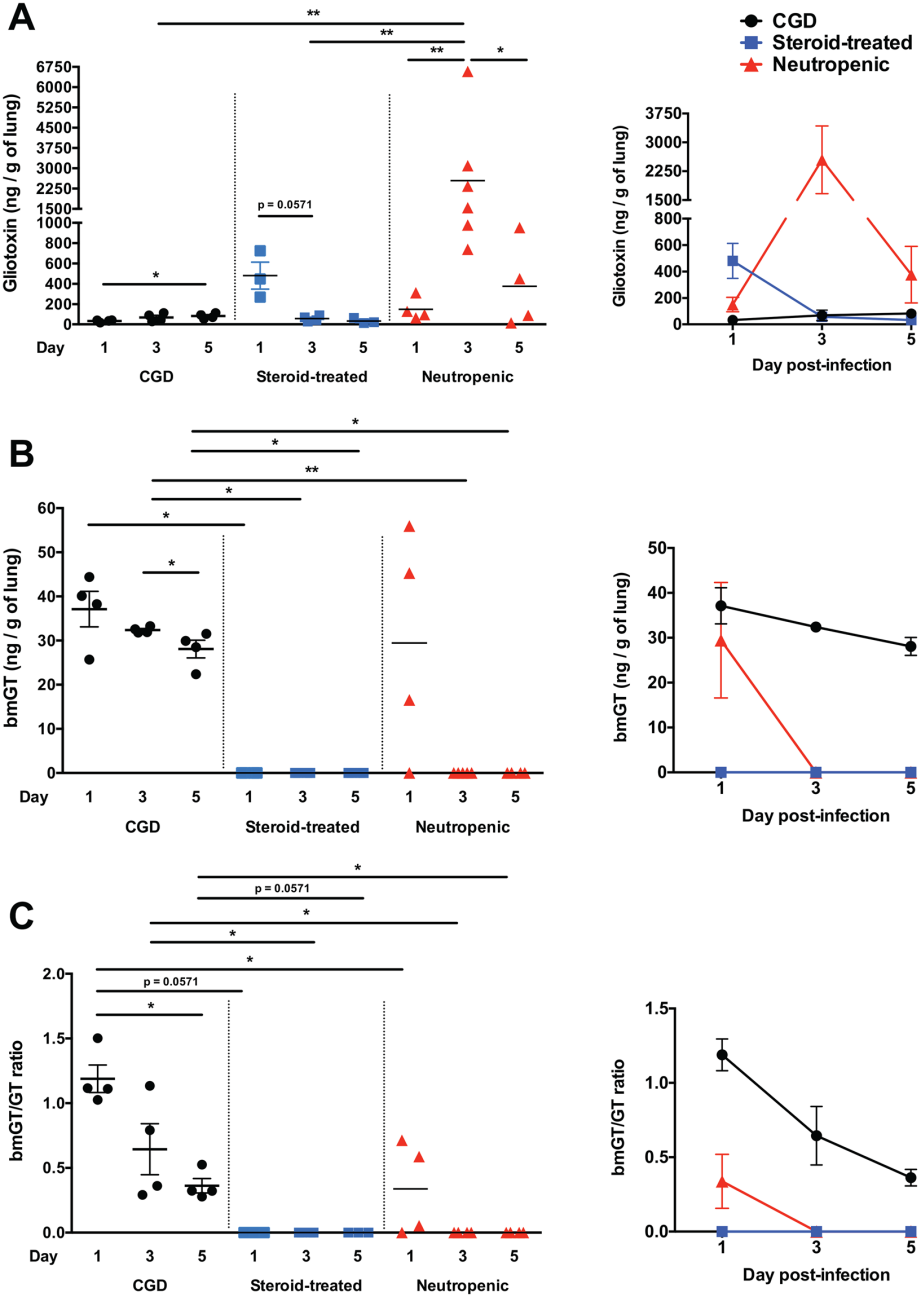
The kinetics of GT and bmGT accumulation in the *A. fumigatus*-infected lung varies depending on the underlying host risk factor. *p47* ^*phox−/−*^ CGD, hydrocortisone-treated, and neutropenic mice were infected with *A. fumigatus* and euthanized at day 1, 3 and 5 post-infection for determination of GT and bmGT levels using HPLC. Shown are levels of GT (**A**), bmGT (**B**) and the bmGT/GT ratio (**C**) in mouse lungs. ^*^ *P* < 0.05; ^**^ *P* < 0.01. Data represent the mean ± SEM. Two independent experiments were performed with similar results with a total of 3–6 mice per time-point.

We infected CGD, steroid-treated, and neutropenic mice with *A. fumigatus*, euthanized them on days 1, 3, and 5 post-infection (Fig. 1A), and harvested lungs, serum and BALF for GT and bmGT analyses using high performance liquid chromatography (HPLC). Although GT was detected in the lungs of all three mouse models examined at all time-points after infection, we observed significant variation in the temporal kinetics of GT production depending on the underlying host risk factor (Fig. 4A). GT levels progressively increased in the CGD lungs during the course of the infection, from a mean of 32.62 ng/g on day 1 (range, 17.09–39.98 ng/g) to a mean of 82.37 ng/g (range, 54.22–113.2 ng/g) of lung on day 5 post-infection. In contrast, GT levels declined during the course of the infection in steroid-treated lungs, from a mean of 481.2 ng/g (range, 269.9–725.6 ng/g) on day 1 to a mean of 31.99 ng/g (range, 12.33–63.08 ng/g) of lung on day 5 post-infection. GT levels peaked on day 3 post-infection in neutropenic lungs at a mean of 2545 ng/g (range, 739.5–6580 ng/g), a statistically significant ~17-fold increase compared to day 1 post-infection (mean, 150.1 ng/g; range, 63.47–312.9 ng/g). In fact, GT levels in neutropenic lungs on day 3 post-infection were ~37.5-fold and ~45-fold greater than those observed in CGD and steroid-treated lungs on day 3 post-infection, respectively. Of potential clinical importance, GT was also detected in 71% of sera and 50% of BALF samples from neutropenic mice on day 3 post-infection, with a decline in 40% of animals on day 5 post-infection, whereas it was undetectable in the serum and BALF throughout the course of IA in both CGD and steroid-treated mice (Table 1).

**Table 1:** Detection of GT and bmGT in sera and BALF during the course of IA.

Model of IA and day post-infection	GT		bmGT	
	Serum	BALF	Serum	BALF
**CGD**				
Day 1	0%	0%	0%	0%
Day 3	0%	0%	0%	0%
Day 5	0%	0%	0%	0%
**Steroid-treated**				
Day 1	0%	0%	0%	0%
Day 3	0%	0%	0%	0%
Day 5	0%	0%	0%	0%
**Neutropenic**				
Day 1	0%	0%	0%	0%
Day 3	71% 55 (14–95)	50% 15 (8–22)	0%	0%
Day 5	40% trace amounts at the limit of detection of the assay (~1 ng/mL)	40% 7 (2–11)	0%	0%

Presented is the % mice with detectable GT or bmGT in serum or BALF samples at the indicated time-point after infection in each mouse model of IA.

Values are in ng/mL of serum or BALF and represent the mean with the range shown in parentheses among positive samples.

As opposed to GT, bmGT was not detected universally in all models of IA, and when detected, its kinetics varied greatly depending on the underlying host risk factor (Fig. 4B). Specifically, bmGT was persistently detected only in CGD lungs throughout the course of IA, with declining levels as the infection progressed, from a mean of 37.12 ng/g (range, 25.68–44.41 ng/g) on day 1 to a mean of 28.08 ng/g (range, 31.82–33.27 ng/g) of lung on day 5 post-infection. Of note, CGD lungs contained greater levels of bmGT relative to GT on day 1 post-infection with a mean bmGT/GT ratio of 1.189 (range, 1.026–1.503), a finding not observed at any other time point or model of infection (Fig. 4C). Of interest, bmGT was not detectable in steroid-treated lungs at any time point after infection, whereas in neutropenic lungs, bmGT was detected only transiently on day 1 post infection with a mean of 29.44 ng/g of lung (range, 0–55.95 ng/g); strikingly, bmGT was undetectable on day 3 post-infection in neutropenic lungs when the levels of GT were at their peak (bmGT/GT ratio of 0; Fig. 4C). bmGT, as opposed to GT, was not detected in the sera or BALF samples of any of the neutropenic mice examined, nor in the sera or BALF samples of steroid-treated and CGD mice (Table 1). Collectively, we found that the temporal kinetics of GT and bmGT accumulation in the *A. fumigatus*-infected lungs is highly dependent on the underlying host risk factor, with GT’s greatest accumulation in neutropenic lungs and bmGT’s greatest accumulation in CGD lungs. Detection of GT in sera or BALF was only observed in neutropenic mice, consonant with extensive peribronchial and vascular invasion of *A. fumigatus* specifically during neutropenic IA (Fig. 3), while bmGT was undetectable in sera or BALF from all *A. fumigatus*-infected mice examined.

### The host specific levels of GT and bmGT correlate with lung fungal burden (in CGD and steroid-treated mice) and hyphal length (in neutropenic mice) but not with the expression of GT/bmGT genes during IA

**Figure 5:**
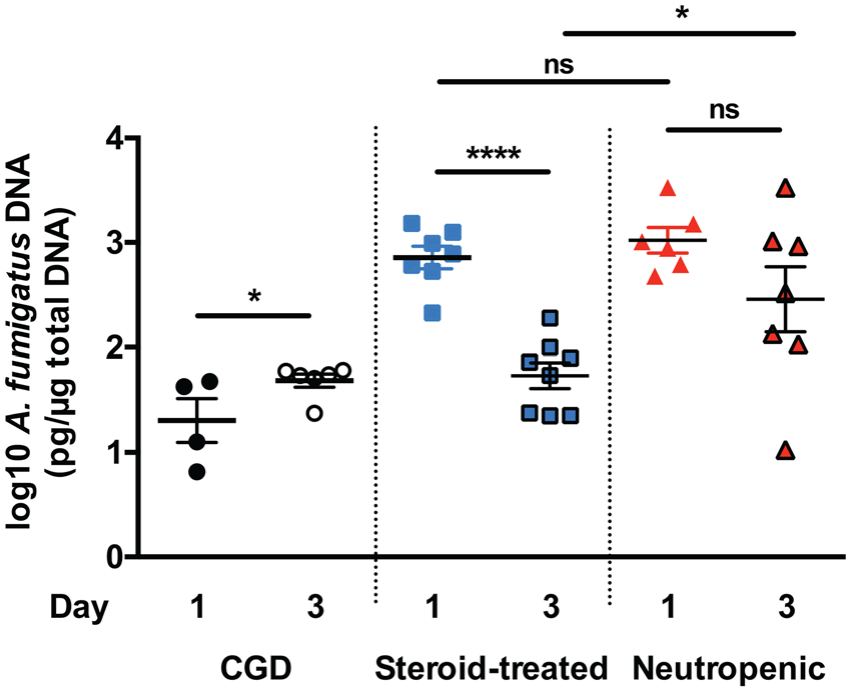
The extent of *A. fumigatus* DNA load in lung tissue during IA. *p47* ^*phox−/−*^ CGD (left panel), hydrocortisone-treated (middle panel), and neutropenic mice (right panel) were infected with *A. fumigatus* and euthanized at day 1 and 3 post-infection for determination of fungal DNA concentration by qPCR. ^*^ *P* < 0.05; ^****^ *P* < 0.0001. ns, not significant. Data represent the mean ± SEM. Two independent experiments were performed with similar results with a total of 4–8 mice per time-point.

We next aimed to examine factors that may account for the observed variation in the temporal dynamics of GT and bmGT levels in the lungs of three mouse models of IA. We first wondered whether this variation reflects a corresponding variation in the temporal kinetics of lung fungal burden as a result of the underlying host risk factor. To investigate this, we focused on days 1 and 3 post-infection, where we observed the greatest host immune status-specific levels of GT and bmGT accumulation in the lung. Infected lung tissue was harvested for quantification of *A. fumigatus* DNA using qPCR, as previously described^29^. Of interest, the extent of lung *A. fumigatus* burden correlated only in a few instances with the observed variations in GT and bmGT levels in the lungs of the three animal models. For example, the decline in lung fungal burden from day 1 to day 3 post-infection correlated with the decline in GT levels in steroid-treated lungs (Figs 4A and 5). In addition, lung *A. fumigatus* burdens were comparable between CGD and steroid-treated mice on day 3 post-infection which mirrored the extent of GT levels in the lungs of these two animal models. In neutropenic mice, however, the extent of GT levels was strikingly discrepant with the lung *A. fumigatus* burden on day 3 post-infection. For instance, while lung *A. fumigatus* burden did not differ in neutropenic mice between days 1 and 3 post-infection, there was a marked increase (~17-fold) in GT lung levels on day 3 post-infection (Figs 4A and 5). Similarly, while the lung fungal burden on day 3 post-infection was ~5-fold greater in neutropenic mice compared to steroid-treated mice, the corresponding GT levels in the neutropenic lung were ~45-fold greater compared to steroid-treated lungs. These findings indicate that other factors besides the extent of lung fungal burden may also contribute to the host-specific variation in GT levels in the lung of three IA models on day 3 post-infection.

**Figure 6:**
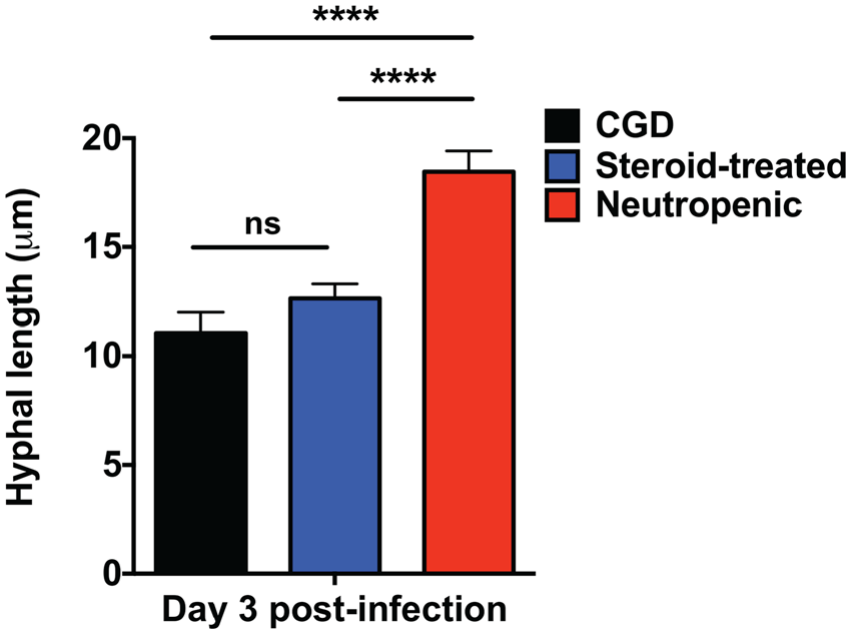
The length of *A. fumigatus* filamentation is greater in neutropenic mice and correlates with the extent of GT accumulation in the lungs. The length of randomly selected individual hyphal elements of *A. fumigatus* in the lung of CGD, steroid-treated and neutropenic mice was measured at day 3 post-infection and found to be greater in neutropenic mice. ^****^ *P* < 0.0001. ns, not significant. Data represent the mean ± SEM. Two independent experiments were performed with similar results with a total of 4–6 mice per time-point and a total of 30–47 randomly selected hyphal elements per mouse group.

Because GT is produced by the hyphal forms of *A. fumigatus* ^14^, we hypothesized that the variation in GT lung levels on day 3 in the three models of IA may reflect host-specific differences in the length of *A*. *fumigatus* hyphal forms produced in the lungs during invasion. This hypothesis was supported by the observation of more extensive *A*. *fumigatus* filamentation in neutropenic lungs by H&E stains (Figs 2 and 3). To examine this further, we analyzed the histopathology slides of GMS-stained lungs on day 3 post-infection and measured the length of randomly selected *A*. f*umigatus* hyphal elements. We found a significantly greater length of *A*. *fumigatus* hyphae in neutropenic lungs, while no difference in hyphal length was observed between CGD and steroid-treated mouse lungs (Fig. 6), indicating that the extent of *A*. *fumigatus* biomass and length of filamentation correlate with the observed kinetics of GT accumulation during IA in the mouse lung.

**Figure 7:**
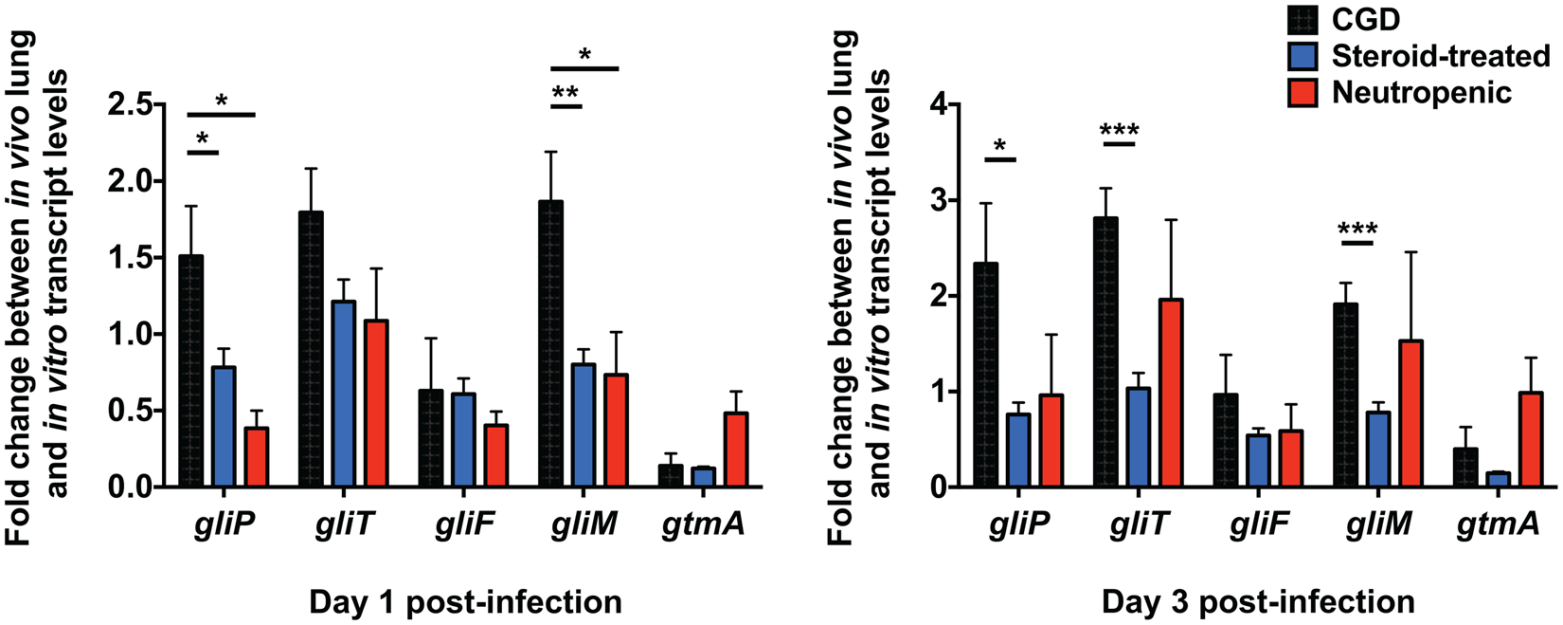
The induction of GT and bmGT biosynthetic genes does not correlate with the host-specific accumulation of GT and bmGT in the mouse models of IA. Shown is the induction of *gtmA*, *gliP*, *gliT*, *gliF*, and *gliM* in the *A. fumigatus*-infected lung for each mouse model, depicted as fold-change in the amount of mRNA of target gene *in vivo* relative to the amount in *in vitro* cultures of *A. fumigatus* (reference standard). ^*^ *P* < 0.05; ^**^ *P* < 0.01; ^***^ *P* < 0.001. Data represent the mean ± SEM. Two independent experiments produced similar results with a total of 4–6 mice per time-point.

We also aimed to gain insight into the host-specific bmGT production in the mouse lung during IA. It was recently demonstrated that S-adenosylmethionine-dependent GT bis-thiomethyltransferase (gtmA) converts dithiogliotoxin to bmGT, and bmGT is a negative regulator of GT biosynthesis upon increase of GT levels *in vitro* cultures of *A. fumigatus* ^21^. Therefore, we postulated that differential *gtmA* induction in the three mouse models of IA may account for the enriched accumulation of bmGT in the CGD lungs, its absence in the steroid-treated lungs, and its transient occurrence in the neutropenic lungs. We carried out transcriptional analyses of *gtmA* as well as of the genes *gliP* (encoding a peptide synthase), *gliT* (encoding gliotoxin oxidoreductase), *gliF* (encoding a cytochrome p450 monooxygenase), and *gliM* (encoding an O-methyltransferase) functioning in the GT biosynthesis pathway on days 1 and 3 post-infection. We found no significant differences in the induction of *gtmA* in the lungs of the three models of IA (Fig. 7). Similarly, induction of *gliF* was comparable in the three models of IA. Instead, induction of *gliP*, *gliT* and *gliM* was enriched in CGD mouse lungs, a finding that did not correlate with higher GT levels relative to steroid-treated or neutropenic lungs. These data underscore the complexity of the transcriptional regulation of GT and bmGT biosynthesis in the infected lung and indicate that other GT/bmGT genes and/or cross-regulatory circuits may be operational *in vivo* relative to *A. fumigatus* cultures *in vitro*. Taken together, these data show that the host-specific variation in GT and bmGT lung levels does not correlate with the degree of induction of GT/bmGT biosynthesis genes at 1 and 3 days post infection with *A. fumigatus*.

## Discussion

Persistent challenges in the timely diagnosis of IA in immunosuppressed patients, coupled with prior data suggesting a potential role for the fungal metabolites GT and bmGT in the pathogenesis and/or detection of IA^15, 18, 19, 22, 23^, led us to design our mouse study. We aimed to assess the diagnostic potential of GT and bmGT for IA among three models of immunosuppression: CGD, steroid-induced, or neutropenia. To our knowledge, this is the first study to date assessing the spatiotemporal kinetics of GT and bmGT in the lung, serum and BALF of immunosuppressed mice following experimental pulmonary *Aspergillus* infection.

Our investigation yielded several important observations: first, we found GT levels to be significantly elevated in the lungs of neutropenic mice relative to the lungs of steroid-treated and CGD mice. On the contrary, bmGT levels were significantly lower than GT levels and only transiently detected in the lungs of neutropenic mice, were not detected in mice with steroid-induced immunosuppression, but were persistently detected in CGD mice at levels comparable to those of GT. The extent of GT levels in lung correlated with both fungal burden and *Aspergillus* hyphal length in tissue. In addition to lung tissue, GT was detected in 71% of sera and 50% of BALF among neutropenic mice while neither GT nor bmGT was detectable in serum or BALF among steroid-treated or CGD mice. Taken together, these findings suggest that in neutropenic mice, in whom there is a greater burden of fungal load as well as vascular and peribronchial invasion, GT may serve as a useful biomarker for IA.

One explanation for the differential detection of GT among the various models of immunosuppression may relate to the model-specific histopathologic changes induced by *Aspergillus* infection. The proximity and tissue burden of *Aspergillus* near the vascular and bronchial trees was most prominent in neutropenic mice, a feature which may increase the likelihood of detecting GT in the blood and bronchial fluid (i.e., serum and BALF). Humans with neutropenia as a risk factor for IA are known to exhibit similar histopathology (i.e., high fungal burden with extensive hyphal growth and vascular/peribronchial invasion) and may thus exhibit similar detection rates for GT. To date, GT/bmGT levels in human fluids have only been assessed via small-scale observational studies. Prospective investigations are warranted to determine the diagnostic value of GT in the serum or BALF of human neutropenic patients with IA. Furthermore, as chemical detection methods evolve beyond HPLC, such as with the use of more sensitive techniques including liquid chromatography–tandem mass spectrometry (LC/MS/MS), the sensitivity of GT as a diagnostic biomarker may be further elucidated.

In our investigations, although bmGT was detectable in the lungs of CGD mice at all time points and was only transiently detectable in the lungs of neutropenic mice (at Day 1 post-infection only), it was not detectable in the sera or BALF in any mouse model at any time point. Thus, bmGT did not appear to be a useful biomarker for the diagnosis of IA in any of the acute mouse models of infection. This observation is in contrast to prior studies in which bmGT (more than GT) has been detected in the serum and BALF of some human patients with IA^22, 23^. The discrepancies between our findings and prior studies of the diagnostic yield of bmGT and GT could result from true differences in mouse versus human infections; for instance, the experimental infection in mice is more acute, with higher fungal burdens than would be expected in human patients. Mice and humans may also differ with respect to biological processing of fungal metabolites *in vivo*. Alternatively, the differential results between mice and humans may reflect incomplete data from the studies of human samples. Prior studies of bmGT detection in human samples have been hampered by small sample sizes and a paucity of cases with a definitive diagnosis of IA (i.e. proven IA as per EORTC / MSG criteria). For instance, in the study by Domingo and colleagues^22^, bmGT was detected in 3 serum samples and 2 BAL samples of patients with probable IA, while no proven cases were examined and the denominator of all tested cases was not clearly defined; in a follow-up, prospective trial^23^, sera from 1 patient with proven IA and 4/5 patients with probable IA were positive for bmGT; neither BALF data nor detailed clinical information as to the patients’ underlying risk factors for IA were reported. In contrast, our investigations using murine models involved direct infection with accompanying histopathologic and microbiologic confirmation, such that results were achievable in a more controlled fashion than could be possible in human studies. Notably, bmGT is recognized to be more stable / less chemically active than GT^22^, which could carry relevance for its utility as a potential biomarker. Certainly, additional studies in humans with probable and proven cases of IA are needed to further define the diagnostic yield of both GT and bmGT from serum and BALF samples in patients with neutropenia and in those with steroid-induced IA.

Besides adding to the literature regarding the potential role of fungal metabolites as diagnostic biomarkers in IA, our investigations also provide insight into the complex biology of GT and bmGT in the setting of pulmonary *Aspergillus* infection *in vivo*. Specifically, we were unable to find a direct correlation between the transcriptional regulation of known GT/bmGT biosynthesis genes at days 1 and 3 post infection and the degree of detectable levels of GT and bmGT in mouse lungs. It is possible that the increase in transcription of the target genes occurred at different timepoints than the timing of our transcriptional analyses. Alternatively, there may be additional, as yet undefined regulatory factors governing the production and processing of GT/bmGT *in vivo*, relative to the *in vitro* culture setting in which these metabolites and regulatory circuits have been previously studied^21^. Therefore, further investigations are needed to better define the factors which drive the synthesis/breakdown and cross-talk between GT and bmGT in the setting of active mammalian infection.

Our study has several limitations. First, all experiments were conducted using a single strain of *Aspergillus fumigatus*. Further studies will be needed to assess whether the temporal kinetics of GT/bmGT detection vary depending on the *Aspergillus fumigatus* strain and/or non-*fumigatus Aspergillus* species. Of note, prior studies have demonstrated lower levels of GT from culture filtrates of non-*fumigatus* species, suggesting a lower diagnostic utility of GT for IA caused by non-*fumigatus Aspergillus* ^30^. Furthermore, assessments of histology and fungal burden, transcriptional regulation of GT/bmGT biosynthesis genes and detection of GT/bmGT levels in lung tissue required separate experiments; we could not feasibly perform all assays as part of a single experiment. Although we employed strict protocols related to experimental infection and immunosuppression for each mouse model and independent experiment, variability between experiments could influence direct assay comparisons. In our study, detection of GT and bmGT in lung, serum and BALF utilized HPLC, for which the limit of detection is at the level of ng/g or ng/mL; it is thus plausible that some of our negative samples could have demonstrated detectable levels of GT and/or bmGT if a more sensitive methodology such as LC/MS/MS was used. Finally, lack of adequate amount of sample precluded simultaneous testing of GM and BDG in the serum or BALF samples; such data would have been helpful in assessing the diagnostic accuracy of GT/bmGT for IA in direct comparison with currently available diagnostic tests. In future studies, the use of LC/MS/MS, which requires lower volumes of serum and BALF for GT/bmGT detection, will enable such comparisons.

In summary, our study assessed the spatiotemporal kinetics of GT and bmGT accumulation in the lungs, sera and BALF of immunosuppressed mice with aspergillosis that mimic the three major risk models for human IA (CGD, steroid treatment and neutropenia). Important findings of our study include the detection of GT in the serum and BALF from the majority of neutropenic mice with IA and the correlation of increased detection with higher fungal burdens and vascular/peribronchial invasion, highlighting the potential for GT as a diagnostic tool for IA in neutropenic patients. Additional prospective studies of human subjects and more mouse studies will further clarify the feasibility and utility of GT and bmGT as diagnostic biomarkers for this morbid condition.

## Methods

### *Aspergillus fumigatus* strain

The clinical *A. fumigatus* isolate B-5233 was maintained as previously described^31^. Freshly harvested conidia were suspended in 0.01% Tween 20 in PBS at various concentrations for mouse infections.

### Mouse models of IA

All animal experiments were carried out under the approval and auspices of the Animal Care and Use Committee of the NIAID at the NIH (protocol LCIM14E) and in accordance with the relevant guidelines and regulations. Sex-matched 6–8 week old mice were used in all experiments. Three experimental models of IA represented the three modes of pathogenesis seen in patients: CGD, corticosteroid-treated, and neutropenic mice. The p47*phox* ^*−/−*^ mice (B6.129S2-Ncf1*tm1Shl* N14) were used for the CGD model. For corticosteroid treatment, we used subcutaneous hydrocortisone injections in C57BL/6 mice as previously described^32^. To induce neutropenia, we injected C57BL/6 mice with 100 μg of Gr-1 monoclonal antibody (clone: RB6-8C5) 16 hours before infection with *A. fumigatus*. This scheme has been shown to result in neutropenia without affecting monocyte/macrophage accumulation in the mouse lung during IA^33, 34^. The mice were inoculated with 30 µl of freshly prepared *A. fumigatus* conidial suspension via pharyngeal aspiration, as previously described^24^. In pilot experiments, the inoculum that results in a LD_80_ between 7–10 days after inoculation was determined and used for each model throughout the study. Specifically, CGD mice received ~3.5 × 10^4^ conidia whereas hydrocortisone-treated WT and neutropenic WT mice received ~6 × 10^6^ conidia.

### Histology

Mice were euthanized at days 1, 3 and 5 post-infection. Lung sections were subjected to histopathologic staining with H&E and GMS and the invasion of fungal elements in peribronchial, interstitial or vascular areas was recorded. For measuring *A. fumigatus* hyphal length, 5–10 photomicrographs were obtained from GMS-stained lung sections of CGD, hydrocortisone-treated, or neutropenic mice at 1, 3 or 5 days post-infection at 40x magnification using a Zeiss microscope fitted with Axiocam camera. Thirty to ~50 randomly selected hyphal elements were measured for each condition and the individual hyphal length was quantified and their peribronchial or interstitial localization was determined using Zen pro imaging software.

### Harvesting of mouse lung tissues, BALF and sera for downstream analysis of GT and bMGT by HPLC

Mice were anesthetized at days 1, 3 and 5 post-infection using ketamine/xylazine. Blood was collected via cardiac puncture and into Z-Gel microtubes (Sarstedt), which were spun at 13,200 rpm for 10 min to harvest serum (typical yield of ~150–250 μl per mouse). The serum was then snap-frozen and kept at −80 °C until analysis. Then, BAL was performed as previously described^33^ using instillation of 1.8 mL of sterile PBS and the BALF (typical yield of ~1.2–1.5 mL per mouse) was frozen and stored at −80 °C until analysis. Then, the lungs were removed aseptically, weighed, and placed in 1.5 mL of PBS containing Tween20 and a protease inhibitor (Roche). After homogenization with Omni Tissue Homogenizer, the supernatant was clarified using a 0.22 μm filter, frozen, and stored at −80 °C until analysis.

### *A. fumigatus* DNA load in mouse lung

Mice were euthanized at days 1 and 3 post-infection. DNA from lungs was isolated using the Fast DNA Spin kit (MPBIO) according to the manufacturer’s protocol. The *A. fumigatus* DNA concentration was determined using qPCR. Primers and probe were specific to the 28S-ITS2 region of the ribosomal subunit gene of *A. fumigatus* ^29^. Two hundred and fifty nanograms of lung’s DNA were used as template. DNA concentration was calculated from a standard curve derived from *A. fumigatus* genomic DNA.

### Transcriptional analyses of *Aspergillus* GT biosynthetic genes in lungs

Transcriptional analysis of the genes *gliP*, *gliT*, *gliF*, *gliM* and *gtmA* from the GT synthesis pathway was carried out using the lungs of mice infected with *A. fumigatus* in the three models of aspergillosis at days 1 and 3 post-infection (*in vivo*) and compared to the levels of the gene expressions from *A. fumigatus* B-5233 *in vitro* cultures (Fig. 7). The *in vivo* versus *in vitro* comparisons were carried out in order to ensure the validity of the transcriptional analysis for each *in vivo* model, since the *in vitro* data served as a reference standard. In other words, presenting the transcriptional data as the ratio of *in vivo*: *in vitro* gene expression facilitated a direct means of comparing gene expression between mouse models given that we could not use uninfected mouse lung tissue for comparison.

*Lungs from mice infected with A. fumigatus:* animals were euthanized and lungs were excised, rinsed with PBS, freeze-dried and RNA was isolated.

*Aspergillus fumigatus B-5233 in vitro sample* *:* 1 × 10^3^ conidia of *A. fumigatus* B-5233 were inoculated onto 100 ml of RPM1640 and incubated for 18 hours on a shaker (220 rpm) at 37 °C; mycelia was harvested, freeze-dried and then RNA was isolated.

*RNA isolation:* Ground samples were treated with Trizol for RNA isolation and RNA was purified with RNeasy Kit (Qiagen) according to manufacturer’s instructions. Isolated RNA was treated with Turbo DNase (Ambion) according to manufacturer’s protocol.

cDNA synthesis: cDNA was synthesized with the High Capacity cDNA Reverse Transcription Kit (Applied Biosystems) according to the manufacturer’s protocol.

*Quantitative real time PCR (qRT-PCR)* *:* assays were carried out as previously described^35^. In brief, 25 ng of cDNA from mice lungs or 1 ng of *A. fumigatus* cDNA were used as template. The *A. fumigatus* glyceraldehyde 3-phosphate dehydrogenase (*gpdA*) gene was amplified as endogenous control to standardize the amount of sample added to the reaction mix. Relative quantitation of gene expression was performed using the relative standard curve method. Standard curves for each primers-probe pairs were performed with cDNA from *A. fumigatus* (0.01 to 10 ng per reaction). Results were expressed as relative fold-change between the transcription of the target gene *in vivo* (lungs) and the transcription *in vitro* (cultured in RPMI 1640). The sequence of the primers and probes used for qRT-PCR were as follows: *gpdA* (forward primer, tctccgctccttctgctgat; reverse primer, cggaggtgtaggtggtgttgt; probe, cccccatgttcgtcatgggtgtc); *gliT* (forward primer, gtgcctcgggactgaaattc; reverse primer, cgacatcgcccttttcga; probe, aggctcgacccatccggcg); *gtmA* (forward primer, gatcgtgaatgcgcttcca; reverse primer, cggatgcgaggatcctttc; probe, ccagacgccaacgccgcc); *gliM* (forward primer, ggcgatttcaccaagcagat; reverse primer, agtcatgcagacaccatttcatg; probe, ccgccgtccgcggtgtaca); *gliF* (forward primer, ggcggcgccaatgag; reverse primer, tgccatggccgaagttg; probe, accgctggcagcatacctcgacg); *gliP* (forward primer, cctgaacgccatgcacaag; reverse primer, ccagccggcggtagaagt; probe, caatccaccttggtcctggccg).

### Quantitative HPLC assay for GT in mouse samples

#### Materials

GT, iodomethane, iodoethane, N,N-diisopropylethylamine, sodium borohydride, tert-butanol were obtained from Sigma-Aldrich, and chloroform was obtained from Macron. HPLC grade solvents were obtained from Burdick & Jackson. WAX and t-C18 microelution solid phase extraction plates were obtained from Waters.

#### Synthesis of bmGT and bisdethiobis(ethylthio)gliotoxin (bEGT)

Five to 10 mg of GT were dissolved in chloroform/methanol, 1/1, to 20 mg/ml. A 3.7 mole excess of N,N-diisopropylethylamine was added. A 2 mole excess of sodium borohydride from a 30 mg/ml ethanol solution was added immediately followed by the addition of a 20 to 25 fold excess of iodomethane or iodoethane. The alkylation was complete within 5 to 8 hours at room temperature as judged by thin layer chromatography (TLC). The solvent was evaporated under vacuum and the residue was dissolved in 3 ml of water. The solution was extracted 3 times with equal volumes of chloroform. The organic phase was evaporated and the residue was dissolved in methanol. The alkylated GT was purified by reverse phase chromatography and the pooled fractions were evaporated under argon. The oily, yellow-orange residue was dissolved in tert-butanol and lyophilized, resulting in a light, white gauzy solid. The final, purified yields were both approximately 30 mole percent. The products were 95% pure as judged by analytical reverse phase HPLC and were single, UV absorbing spots on silica gel TLC developed in dichloromethane/methanol, 97/3 and toluene/ethyl acetate/formic acid 5/4/1. Both compounds were stable for at least two years in methanol at −20 °C. Analysis using a 4000 QTrap mass spectrometer with a spray solvent of 70% acetonitrile, 0.1% formic acid, revealed the expected, positively charged masses of 357 and 387 amu for bmGT and bEGT, respectively. Their sodium adducts were also observed. The in-source loss of sulfur is typically seen with GT in electrospray conditions and loss of methanethiol and ethanethiol groups with the alkylated derivatives were also observed. The molar extinctions in ethanol at 270 nm of bmGT and bEGT were calculated to be 4100 (average of three syntheses). The molar extinction of GT at 270 nm in ethanol was taken as 4500^36^.

#### Sample Preparation

A. BALF: 0.75 ml of BALF sample was centrifuged at 16,000 g for 5 min at 4 °C to remove particulates, cell debris and mucous after thawing. 30 ng of bEGT (10 μl of PBS 3 ng/μl) was added per sample and the samples were loaded at approximately −1 mm Hg on a t-C18 SepPak microelution plate equilibrated in 10% methanol. The wells were washed with 0.2 ml 10% methanol and eluted with 0.25 ml 80% methanol. The eluted samples were dried under vacuum, dissolved in 5 μl 40% methanol, centrifuged at 16000 g at 4 °C for 5 min and transferred to autosampler vials.

B. Serum: Serum samples were centrifuged as described to clear additional clots and precipitated material after thawing. Typically, 0.1 to 0.2 ml of serum was sampled. All final volumes were made to 0.25 ml with PBS. Thirty ng of bEGT was added and the samples were applied to the t-C18 plates, washed with 0.2 ml 10% methanol and eluted with 0.25 ml of 50 mM ammonium acetate, pH 6.0, 80% methanol. The samples were loaded on Oasis WAX microelution plates equilibrated in the same solution. The flow through was collected, dried under vacuum and prepared as described for the BALF samples. This step eliminated several interfering peaks in the chromatography.

C. Lung homogenates: Thawed samples were cleared by centrifugation. Protein concentration was determined by the bicinchoninic acid (BCA) assay. Thirty ng of bEGT was added to each sample, which were then loaded on the t-C18 plates, washed and eluted with 80% methanol. The samples were dried under vacuum and dissolved in 0.25 ml 50 mM ammonium acetate, pH 6.0, 50% methanol, loaded on to Oasis WAX microelution plates equilibrated in the same solution and the flow through collected. The samples were dried and dissolved as described.

#### HPLC analysis

Samples were analyzed by chromatography on an Agilent 1100 Capillary HPLC system with diode array detection using a 0.5 mm × 150 mm Jupiter Proteo reverse phase column (Phenomenex) at a flow rate of 12 μl per min. Two microliters of sample were injected and the column was developed isocratically for 20 min with a solvent mixture of 60% water and 40% methanol. A gradient to 80% methanol was then run for 20 min. GT was eluted isocratically at approximately 19 min, bmGT and bEGT were eluted at approximately 52% and 78% methanol, respectively. Detection and peak integration were performed at 270 nm and spectral acquisition was made between 200 nm and 300 nm. The sensitivity for the three compounds was 0.5 ng with pure standards and 2 ng to 4 ng with standards spiked in negative sample matrices. The linear dynamic range for all three compounds was 1 ng to 200 ng. Over the selected working range of 1 ng to 50 ng of GT and bmGT the response ratios to 30 ng of the bEGT internal standard was linear with correlations 0.992 to 0.996. Recoveries of 100 ng of spiked GT and bmGT standards in the BALF assay were 61% and 77% with relative recoveries to standard of 81% and 100% respectively. GT concentrations were corrected for this difference in relative recoveries. The serum assay showed recoveries of GT and bmGT of 75% and 80% with relative recoveries of 96% and 99% respectively. Lung homogenates showed recoveries of GT and bmGT of 70% and 78% with relative recoveries of 93% and 100%. Importantly, for HPLC data validation purposes, we did not detect GT and bmGT by HPLC in uninfected mouse samples (Figure S1). In addition, the chromatogram features and PDA (photodiode array) spectra of GT and bmGT were similar in mouse samples after *Aspergillus* infection and in uninfected mouse samples spiked with pure GT and bmGT standards (Figure S1 and data not shown). Last, while we did not have access to LC-MS for analysis of all of our experimental samples, we used LC-MS during the development of our HPLC assay and we confirmed by LC-MS the GT detection in mouse samples by HPLC (data not shown).

### Statistics

The experimental data were analyzed using unpaired *t*-test or Mann-Whitney test as appropriate with GraphPad Prism 6.0 and presented as mean values ± SEM. Statistical significance was defined as *P* < 0.05.

### Data Availability Statement

The datasets generated during and/or analysed during the current study are available from the corresponding author on reasonable request.

## Electronic supplementary material

Supplementary Information
