# Supplementary material for: Host immune status-specific production of gliotoxin and bis-methyl-gliotoxin during invasive aspergillosis in mice
Source: Sci Rep. 2017 Sep 8;7:10977. doi: 10.1038/s41598-017-10888-9 (PMC5591180; doi:10.1038/s41598-017-10888-9)

Supplementary Information:

Host immune status-specific production of gliotoxin and bis-methyl-gliotoxin during invasive aspergillosis in mice

Janyce A. Sugui[*1], Stacey R. Rose[2*§], Glenn Nardone[3], Muthulekha Swamydas[2], Chyi-Chia R. Lee[4], Kyung J. Kwon-Chung[1¶], Michail S. Lionakis[2¶]

[*]These authors contributed equally to this work.

[¶]Corresponding authors:

lionakism@mail.nih.gov; jkchung@niaid.nih.gov

Authors' affiliations:

[1]Molecular Microbiology Section, Laboratory of Clinical Immunology & Microbiology (LCIM), National Institute of Allergy & Infectious Diseases (NIAID), National Institutes of Health (NIH), Bethesda, MD, USA; [2]Fungal Pathogenesis Unit, LCIM, NIAID, NIH, Bethesda, MD, USA; [3]Research Technology Branch, NIH, Rockville, MD, USA; [4]Laboratory of Pathology, Center for Cancer Research, National Cancer Institute (NCI), NIH, Bethesda, MD, USA.

[§]currently at the Division of Infectious Diseases, Baylor College of Medicine, Houston, Texas, USA.

Figure S1 Legend. HPLC chromatograms of GT and bmGT from *Aspergillus*-infected lung tissue. Shown are representative HPLC chromatograms that show absence of GT or bmGT in an uninfected mouse lung tissue homogenate (panel A), detection of GT (red arrow) and bmGT (black arrow) in an *Aspergillus*-infected mouse lung homogenate (panel B), and detection of GT (red arrow) and bmGT (black arrow) in an uninfected mouse lung tissue homogenate that has been spiked with pure GT and bmGT standard (panel C).

Figure S1

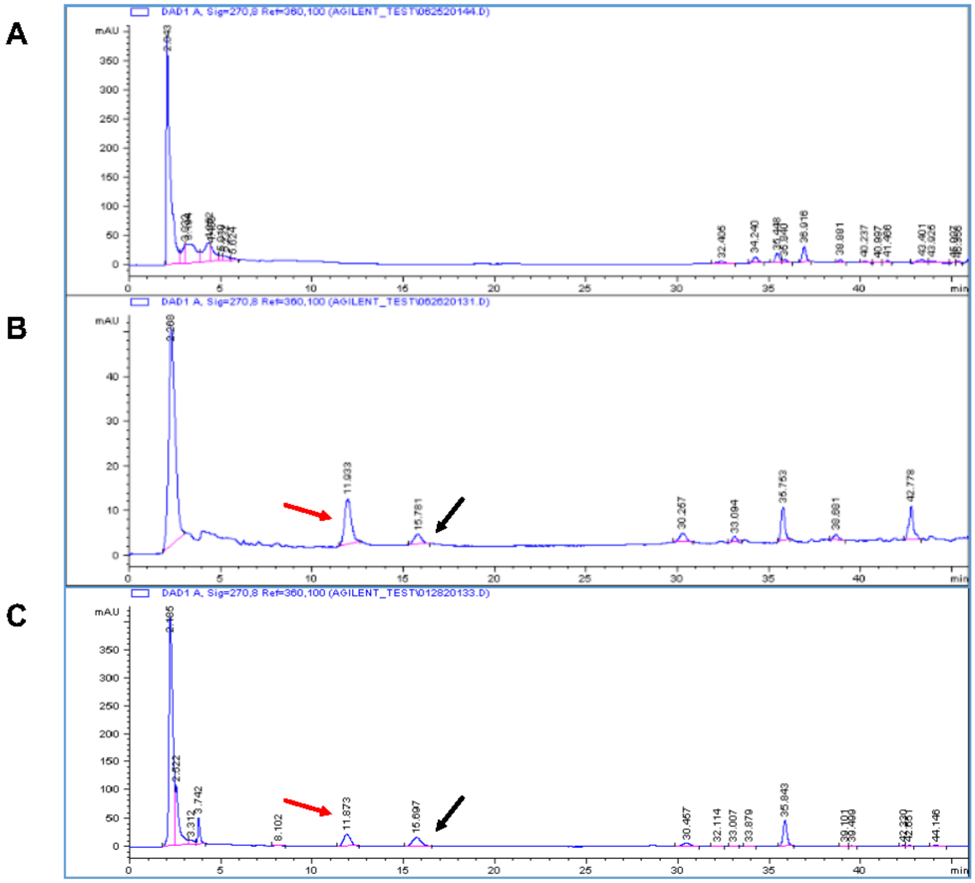

Supplement: Supplementary file 1 — Supplementary Information [file 41598_2017_10888_MOESM1_ESM.pdf]
